# Human Adipose-Derived Mesenchymal Stem Cells as a New Model of Spinal and Bulbar Muscular Atrophy

**DOI:** 10.1371/journal.pone.0112746

**Published:** 2014-11-13

**Authors:** Marta Dossena, Gloria Bedini, Paola Rusmini, Elisa Giorgetti, Alessandra Canazza, Valentina Tosetti, Ettore Salsano, Anna Sagnelli, Caterina Mariotti, Cinzia Gellera, Stefania Elena Navone, Giovanni Marfia, Giulio Alessandri, Fabio Corsi, Eugenio Agostino Parati, Davide Pareyson, Angelo Poletti

**Affiliations:** 1 Cellular Neurobiology Laboratory, Unit of Cerebrovascular Disease, Fondazione IRCCS Istituto Neurologico Carlo Besta, Milan, Italy; 2 Dipartimento di Scienze Farmacologiche e Biomolecolari, Centro Interdipartimentale sulle Malattie Neurodegenerative, Università degli Studi di Milano, Milan, Italy; 3 Department of Pathology, University of Michigan, Ann Arbor, Michigan, 48109, United States of America; 4 Clinic of Central and Peripheral Degenerative Neuropathies Unit, Department of Clinical Neurosciences, Fondazione IRCCS Istituto Neurologico Carlo Besta, Milan, Italy; 5 Unit of Genetics of Neurodegenerative and Metabolic Diseases, Department of Diagnostic and Applied Technology, Fondazione IRCCS Istituto Neurologico Carlo Besta, Milan, Italy; 6 Surgery Division, Department of Clinical Sciences, University of Milan, “Luigi Sacco” Hospital, Milan, Italy; Children's Hospital of Pittsburgh, University of Pittsburgh Medical Center, United States of America

## Abstract

Spinal and bulbar muscular atrophy (SBMA) or Kennedy's disease is an X-linked CAG/polyglutamine expansion motoneuron disease, in which an elongated polyglutamine tract (polyQ) in the N-terminal androgen receptor (ARpolyQ) confers toxicity to this protein. Typical markers of SBMA disease are ARpolyQ intranuclear inclusions. These are generated after the ARpolyQ binds to its endogenous ligands, which promotes AR release from chaperones, activation and nuclear translocation, but also cell toxicity. The SBMA mouse models developed so far, and used in preclinical studies, all contain an expanded CAG repeat significantly longer than that of SBMA patients. Here, we propose the use of SBMA patients adipose-derived mesenchymal stem cells (MSCs) as a new human *in vitro* model to study ARpolyQ toxicity. These cells have the advantage to express only ARpolyQ, and not the wild type AR allele. Therefore, we isolated and characterized adipose-derived MSCs from three SBMA patients (ADSC from Kennedy's patients, ADSCK) and three control volunteers (ADSCs). We found that both ADSCs and ADSCKs express mesenchymal antigens, even if only ADSCs can differentiate into the three typical cell lineages (adipocytes, chondrocytes and osteocytes), whereas ADSCKs, from SBMA patients, showed a lower growth potential and differentiated only into adipocyte. Moreover, analysing AR expression on our mesenchymal cultures we found lower levels in all ADSCKs than ADSCs, possibly related to negative pressures exerted by toxic ARpolyQ in ADSCKs. In addition, with proteasome inhibition the ARpolyQ levels increased specifically in ADSCKs, inducing the formation of HSP70 and ubiquitin positive nuclear ARpolyQ inclusions. Considering all of this evidence, SBMA patients adipose-derived MSCs cultures should be considered an innovative *in vitro* human model to understand the molecular mechanisms of ARpolyQ toxicity and to test novel therapeutic approaches in SBMA.

## Introduction

Spinal and bulbar muscular atrophy (SBMA) or Kennedy's disease, an X-linked disorder affecting adult males, is characterized by wasting and weakness of facial, bulbar and limb muscles associated with motoneuron degeneration in brainstem and spinal cord. Mild sensory signs occur related to abnormalities of dorsal root ganglia neurons [1]. Muscle atrophy results from both denervation and direct involvement of muscle cells [2]. Signs of androgen insensitivity (gynecomastia, hypogonadism, and reduced fertility) can be also observed. No treatment or cure for SBMA is available.

SBMA is linked to a CAG repeat expansion in the androgen receptor (AR) gene, which is translated into an elongated polyglutamine tract (polyQ) in the AR protein (ARpolyQ) [3]. The ARpolyQ alters AR behaviour, conferring neurotoxicity responsible for motoneuron death [3]–[5]. In fact, the polyQ induces AR misfolding and its aggregation into cytoplasmic and nuclear inclusions. This is triggered by testosterone and dihydrotestosterone, which activate AR [6]–[8] inducing the AR nuclear neurotoxicity [9], [10].

Different SBMA mouse models have been developed and used in preclinical studies until now, which demonstrated the prominent role of androgens in symptoms appearance, disease progression and death. These mice have been generated using a CAG repeat of a size markedly higher than that found in the human disease [10]–[16]. In addition, in most mouse models the AR transgene expression is driven by constitutive promoters (such as actin or prion promoters), with the only exception of a knock-in SBMA mouse model, in which ARpolyQ expression is driven by endogenous promoter to maintain normal AR synthesis and localization. Alternative SBMA mice models have been developed using a human AR promoter by using either YAC or BAC constructs to insert the entire human AR gene. Despite of being under the control of an “exogenous” promoter, and the possible differences in transcriptional regulation between species, these mice should also mimic the tissue distribution of the AR protein found in human [12], [13], [17]. However, the use of longer AR CAG repeats dramatically accelerates the disease phenotype in these SBMA animal models, which instead is normally characterized by a very slow progression rate in patients. This aspect has not been taken into account in all murine models [18]. Therefore, it is important to develop a new model closer to human pathological condition to test innovative drug treatments designed to reduce cytotoxic aggregates.

Induced pluripotent stem cells (iPSCs) have been recently developed from SBMA patients. Their relevant value is to be cells of human origin that can be successfully differentiated toward a motoneuronal phenotype, to produce reliable cell models that mimic disease in this particular cell type affected in SBMA [19]. However, muscle tissue is another target of ARpolyQ toxicity, and, to the best of our knowledge, all attempts to generate muscle cells from iPSCs failed so far. In addition, iPSCs are produced by genetic transformation of fibroblasts, using four oncogenic or differentiating agents that may impact on cell behavior. Thus, other cell models of human origin may be of value to complement the data obtained in iPSCs.

Mesenchymal stem cells (MSCs), originally identified in bone marrow stroma, can be isolated from different tissues (e.g.: umbilical cord blood, adipose tissue), expanded and differentiated *ex vivo* into multiple cell types [20]. Moreover, compared to iPSCs, MSCs are not retro-induced with genes involved in oncogenic cell transformation.

Adipose tissue is an abundant, accessible source of adipose-derived MSCs (ADSCs) [20], which contains a population of mesenchymal stem cells with no tumorigenic or telomerase activities [21], [22], with marked neuro-immunomodulatory properties and the capability to migrate to sites of injury, thereby conferring them as a possible contributor in tissue repair. AR is highly expressed in adipose tissue [21], [23], where androgens modulate ADSCs commitment to pre-adipocytes [21], [24]. Moreover, ADSCs from SBMA patients present the advantage that they express only the ARpolyQ, and not the wild type allele. Hence, we evaluated the potential of ADSCs to be used as novel human SBMA *in vitro* models to better understand ARpolyQ-toxicity with the overall goal of finding novel future therapeutic approaches.

We found that AR is highly expressed in normal ADSCs while it was reduced in SBMA ADSCs. Testosterone induced AR nuclear translocation and, in a limited SBMA cellular population, AR nuclear inclusions after proteasome inhibition. Therefore, ADSCs could be considered as an innovative SBMA human model useful for clarifying molecular mechanisms underlying SBMA pathophysiology.

## Materials and Methods

### Cell isolation and culture

The study was approved by the local institutional review board of the Fondazione IRCCS Istituto Neurologico “C. Besta” (Milan, Italy). Informed written consent was obtained from all volunteers and SBMA patients. The study conformed with the 2013 WMA Declaration of Helsinki.

Specimens of fat from periumbilical regions of three male controls undergoing surgery for ventriculoperitoneal shunt (ADSC samples) (aged 55, 69, 73 years; specimen CAG repeat number = 22, 23, 24) and three SBMA patients (ADSC Kennedy, ADSCK, samples) (aged 49, 57, 76 years; leukocyte CAG repeat number = 46, 44, 44), were mechanically dissociated, washed in PBS 1X (EuroClone, Milan, Italy) and centrifuged at 1300×g for 10 min; the upper phase was plated into T75-cm^2^ flasks, allowed to dry and then Stem Cells Medium was added [SCM: DMEM-F12 with 10% Fetal Bovine Serum (Gibco, Grand Island, NY, USA), 1% penicillin/streptomycin solution (Sigma-Aldrich, Basel, Switzerland)] [25]. Cells were seeded in T75-cm^2^ flasks at 1×10^4^ cells/cm^2^, and passed weekly, for expansion or freezing procedures. Freezing was performed in FBS with 10% of dimethyl sulfoxide (DMSO) (–80°C freezer for 24 hours, then stored in liquid nitrogen). After de-freezing by quickly thawing at 37°C, cells were plated in T75-cm^2^ flasks with SCM for 24 hours, and grown in fresh SCM. For the experiments, cells were used before passage nine. Cell viability was assessed by Trypan Blue dye exclusion assay (EuroClone).

### Growth curve

Cell growth was analysed by direct cell counts and by calculation of cumulative population doublings at each passage (three to nine) with the formula:

log_10_ (harvested cells/seeded cells)/log_10_ (2) [26].

Cells (2×10^5^) were seeded in a T25-cm^2^ flask with 3.5 ml of SCM. After 4 days of culture, cells were harvested, counted and re-seeded for next passage growth.

### Flow cytometry analysis

Flow cytometry (FC) was performed to evaluate the mesenchymal phenotype: CD105 (AbDSerotec, Raleigh, NC, USA), CD90 (Millipore Temecula, CA, USA), CD73, CD14, CD19, CD31, CD34, CD45, HLA-DR (BD Pharmingen, San Jose, CA, USA). Briefly, 10^5^ cells/tube were stained with fluorochrome conjugated monoclonal antibodies for 30 min at 4°C in the dark. After centrifugation at 1300×g for 10 min and a PBS wash, cells were fixed with 4% paraformaldehyde (Sigma Aldrich). Fluorescence-activated cell sorting was performed with Cell Quest software (BD Pharmingen). Non-viable cells were excluded according to the side scatter vs. forward scatter parameters, and 5,000 events were acquired for each sample.

### Multipotency characterization of ADSCs

ADSCs and ADSCKs were tested for their ability to differentiate into adipocytes, chondrocytes and osteocytes using Human Mesenchymal Stem Cell Functional Identification Kit (R&D Systems), according to the manufacturer instructions. Adipogenic differentiation was performed starting from 2.1×10^4^ cells/cm^2^ in SCM up to confluence, when SCM was replaced with Adipogenic Differentiation Medium (and changed every 3–4 days). After 21 days, cells were fixed with 4% paraformaldehyde for 1 hour and visualized by Oil Red O Staining (Sigma Aldrich).

Chondrogenic differentiation was performed with 2.5×10^5^ cells seeded in 15 ml conical tubes in Chondrogenic Differentiation Medium (replaced every 2–3 days); after 21 days, chondrocyte pellet was fixed with 4% paraformaldehyde for 20 min and immunostained for aggrecan (R&D System).

Osteogenic differentiation was done starting form 4.2×10^3^ cells/cm^2^ in SCM up to 70–80% confluence, when SCM was replaced with Osteogenic Differentiation Medium (changed every 3–4 days). After 21 days, cells were fixed 10 min with 70% ethanol and processed for Alizarian Red Staining (Sigma Aldrich). Images were obtained at 20X magnification, using Nikon Eclipse TE300 equipped with the Axiovision device camera (Zeiss Instr., Oberkochen, Germany). Images were processed using Axiovision release 4.6.3 (Zeiss Instr., Oberkochen, Germany).

### Immunofluorescence analysis

Cells (4000 cell/mL) plated on glass coverslips in 24-well multiwell plates in SCM with 10% charcoal stripped-FBS (CS-FBS; basal condition to eliminate endogenous steroids) were grown to confluence in absence (–T) or presence (+T) of 10 nM of testosterone (T) for 48 hours, with or without a proteasome inhibitor (MG132, 10 µM; Sigma-Aldrich, St Louis, MO, USA) for 24 hours. Cells were fixed using 4% paraformaldehyde and AR detected using an anti-AR rabbit antibody (D6F11, Cell Signaling Technology, Inc., Danvers, MA, USA) 1∶200 in milk followed by Alexa 488 anti-rabbit (Molecular Probes), 1∶1,000 in milk. Double immunofluorescence (IF) analyses were done with H280 anti-AR (H280) (Santa Cruz Biotech, SantaCruz, CA, USA) 1∶100 in milk with a) mouse monoclonal Hsp70 (sc-24, Santa Cruz Biotech) or b) mouse monoclonal anti-Ubiquitin (sc-8017, Santa Cruz Biotech) 1∶100 in milk, followed by Alexa 488 anti-rabbit (to visualize AR) or Alexa 594 anti-mouse (Molecular Probes, to visualize Hsp70 or Ubiquitin) (1∶1,000 in milk).

Nuclei were visualized with DAPI. Images were obtained at 63X magnification, using an Axiovert 200 microscope (Zeiss Instr., Oberkochen, Germany) with Photometric Cool-Snap CCD camera (Ropper Scientific, Trenton, NJ, USA). Images were processed using Metamorph software version 7.7.7.0 (Universal Imaging, Downingtown, PA, USA).

### Western blot analysis

Western blot analysis (WB) was performed as previously described [27]. Cells were grown in SCM with 10% CS-FBS in the absence (–T) or in the presence (+T) of 10 nM of testosterone (T) for 48 hours, then harvested, centrifuged 10 min at 1300×g. Pellets were resuspended in 150 µl RIPA buffer containing protease inhibitors cocktail (Sigma-Aldrich) homogenized using slight sonication and total protein concentration determined with bicinchoninic acid kit (BCA assay, Thermo Scientific Pierce, IL, USA). WB was performed using 10% SDS-PAGE with 15 µg total proteins. Electrotransfer on nitrocellulose membrane was done with Transblot Turbo Transfer System (Bio-Rad). Membranes were treated with 5% nonfat dried milk powder (Euroclone, Italy) in Tween-TBS (TBS-T, 20 mM TrisHCl, pH 7.5, 0.5 M NaCl, 0.05% Tween-20) for 1 hour and incubated with the following primary antibodies: (a) rabbit polyclonal AR-H280 (Santa Cruz, 1∶1,000) to detect wtAR and ARpolyQ; (b) mouse monoclonal anti-α-tubulin (Sigma Aldrich, 1∶3,000). The following secondary peroxidase-conjugated antibodies were used: goat anti-rabbit to identify the anti-AR (sc-2004, Santa Cruz, dilution 1∶5,000) and goat anti-mouse to identify the anti-α-tubulin (sc-2005, Santa Cruz, dilution 1∶5,000). The immunoreactivity was visualized with enhanced chemiluminescence detection kit (Amersham ECL Prime Western Blotting Detection Reagent). WB images were obtained with ChemiDoc XRS System (Bio-Rad).

### mRNA expression analysis

For real-time PCR (RT-qPCR), cells were plated into T75-cm^2^ flasks in SMC+10% FBS. Total RNA was isolated with TRIreagent protocol (Sigma-Aldrich) as previously described [27]. Total RNA (1 µg), treated with DNAse, was reverse-transcribed into cDNA using High-Capacity cDNA Archive Kit (Applied Biosystems).

RT-qPCR for human AR and GAPDH mRNAs were designed using the program Primer Express 3 and synthesized by MWGBiotech (Ebersberg, Germany) with the following sequences: hARforward: 5′-ATCCCAGTCCCACTTGTGTC-3′; hARreverse: 5′-GGTCTTCTGGGGTGGAAAGT-3′; hGAPDHforward: 5′-GAAGGTGAAGGTCGGAGTC-3′, hGAPDHreverse: 5′-TTGATGGCAACAATATCCACTT-3′. Primer efficiencies was close to 100% for both target and reference gene. RT-qPCR was performed using CFX 96 Real Time System (Bio-Rad) in a 10 µl total volume with iTaq SYBR Green Supermix (Bio-Rad), and 500 nmol primers, in the following conditions: 94°C for 10 min, 35 cycles at 94°C for 15 s and 60°C for 1 min. Melting curve analysis was always performed at the end of each PCR assay to control specificity. Data were expressed as Ct values and used for relative quantification of targets with ΔΔCt calculation. Potential bias, due to averaging data transformed through the equation 2^−ΔΔCt^ to give N-fold changes in gene expression, were excluded performing all statistics with ΔCt values, and hAR values normalized with hGAPDH values.

### Statistical analysis

Statistical analysis was performed using one-way analysis of variance (ANOVA) for group comparisons followed by Bonferroni *post hoc* test, using PRISM software (GraphPad, San Diego, CA, USA). Data were expressed as mean±SD or mean±SEM of three independent samples. P<0.05 was considered statistically significant.

## Results

### ADSCs and ADSCKs characterization

**Figure 1:**
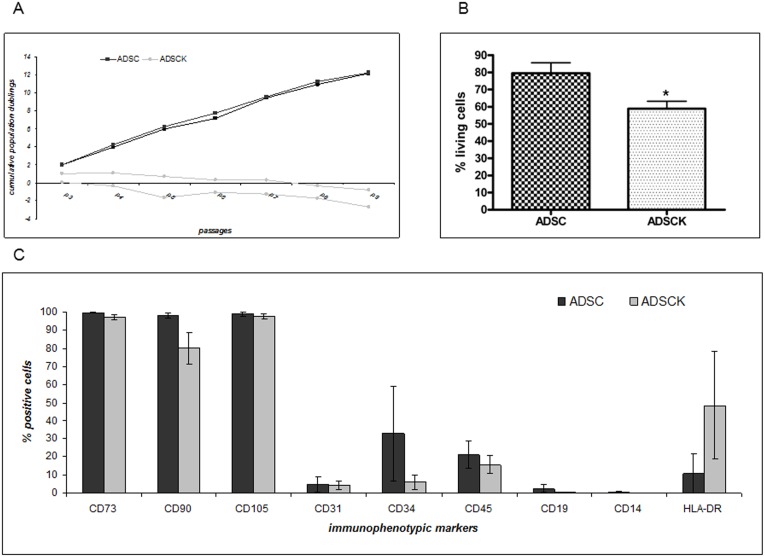
Characterization of ADSC and ADSCK cell cultures. (A) Growth curve of two representative ADSC and ADSCK primary cultures. Values were expressed as cumulative population doublings calculated with the formula reported by Avanzini et al. [26], [44]. (B) Cell viability after freezing and thawing evaluated by trypan blue dye exclusion assay comparing the number of viable cells after thawing to the number of cells previously frozen. Data are expressed as mean±SEM (n = 9 per group; three independent experiments for each cell culture; *P<0.05 vs ADSCs). (C) FACs analysis performed at passage 3 on ADSC (black bar) and on ADSCK (grey bar) to evaluate immunophenotypically mesenchymal markers. Data are expressed as mean±SD (n = 3 per group).

ADSCs derived from fat specimens of the periumbilical regions of controls (n = 3, ADSCs) and SBMA patients (n = 3, ADSCKs) were characterized by evaluating their growth in adhesion and by direct cell count resulting in the cumulative population doubling [26]. The analyses (Figure 1A) show that the two cell populations have different growth rates since, at passage 3, the mean cumulative population doubling value was 1.74±0.41 for ADSCs and 0.5±0.6 for ADSCKs, confirmed also at advanced passages. Moreover, after thawing the viability measured with Trypan Blue exclusion dye assay (Figure 1B) was 79.43±6.12% for ADSCs and only 58.85±4.30% for ADSCKs.

Immunophenotypic analysis, performed on cells collected at passage 3 (Figure 1C), showed that both cell cultures (n = 3, ADSCs; n = 3, ADSCKs) express mesenchymal markers with some differences. ADSCs were positive for mesenchymal antigens CD73 (99.6±0.2%), CD90 (98.1±1.4%) and CD105 (99.0±1.2%), negative for lymphocytic markers CD14 (0.5±0.6%) and CD45 (21.0±7.6%); ADSCs were slightly positive for early hematopoietic marker CD34 (32.8±26.3%) and negative for CD19 (2.5±2.2%), for endothelial typical marker CD31 (4.8±4.1%), and for immunological marker HLA-DR (10.6±10.9%). ADSCKs were positive for CD73 (97.2±1.4%), CD90 (80.1±8.6%) and CD105 (97.6±1.3%) and negative for CD14 (0.1±0.0%), CD45 (15.6±5.0%), CD34 (6.0±4.1%), CD19 (0.3±0.1%), CD31 (4.2±2.6%); only the immunological marker HLA-DR was slightly higher than expected (48.4±29.9%).

Immunophenotypic characterization performed after repeated passages (passage 8), showed that all the three different cultures of ADSCs preserved mesenchymal markers expression CD73 (99.2±0.5%), CD90 (98.9±1.2%) and CD105 (98.6±2.1%), remaining negative for lymphocytic (CD14 0.4±0.2%; CD45 5.2±3.3%), early hematopoietic (CD34 19.0±13.7%; CD19 2.2±1.91%), endothelial (CD31 3.9±4.0%) and immunological (HLA-DR 9.0±8.7%) markers. The three different cultures of ADSCKs were not analysed because of their reduced viability at high passages (see Figure 1A).

### Multipotent ability of ADSCs and ADSCKs

**Figure 2:**
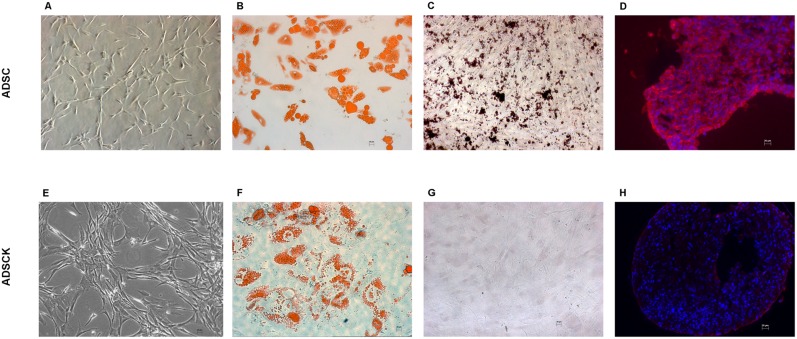
Multipotency characterization of ADSCs and ADSCKs. (A–E) Representative images of normal morphology in absence of differentiation stimuli. (B–F) Adipogenic differentiation was confirmed by Oil Red O Staining. (C–G) Osteogenic differentiation was displayed by Alizarin Red staining. (D–H) Chondrogenic differentiation was visualized by Aggrecan immunofluorescence (red); nuclei were stained with DAPI (blue). (20X magnification).

In undifferentiated conditions, ADSCs and ADSCKs were plastic adherent with typical small, spindle-shape morphology (Figure 2A). When induced to differentiate into adipocytes, osteocytes and chondrocytes (passage 4), they lost their typical aspect. In particular, ADSCs differentiated in all three cell lineages, forming lipid-droplets stained with Oil Red O (Figure 2B) typical of adipocytes, calcium deposition stained with Alzarin Red (Figure 2C), usually present in osteoblasts, and expressing aggrecan (Figure 2D) a typical marker specific for chondroblast cell lineage. Conversely, ADSCKs differentiated only into adipocytes (Figure 2F), since cells stained were positive for lipid-droplets (Oil Red O), but negative for calcium deposits (Figure 2G) and aggrecan expression (Figure 2H).

### Characterization of AR expression and its effect on ADSCs and ADSCKs growth and survival

**Figure 3:**
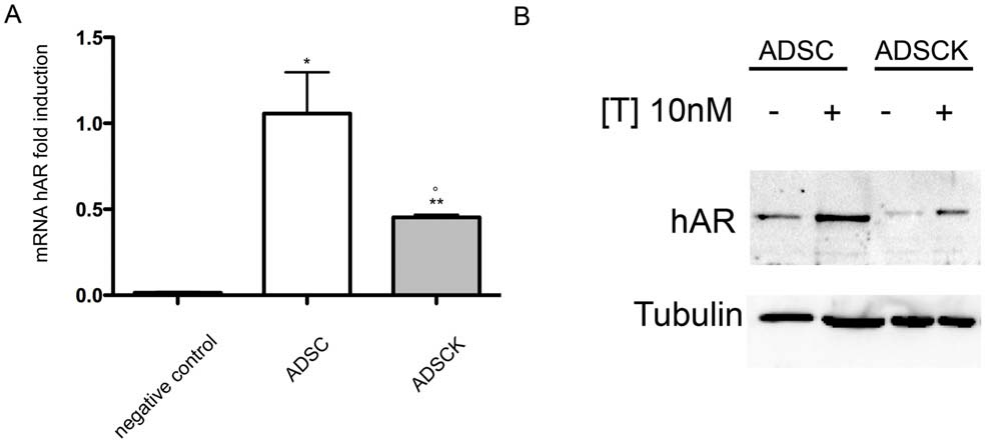
Characterization of AR expression in ADSCs and ADSCKs. (A) AR mRNA levels in ADSCs and ADSCKs were determined by real time quantitative PCR. SHSY-5Y cells were used as negative control. (n = 3 per group; *P<0.05, **P<0.01 *vs* negative control; °P<0.05 *vs* ADSCs). (B) Western Blot on ADSCs and ADSCKs in absence (–T) or in presence (+T) of 10 nM of testosterone for 48 hours. Alpha-tubulin was used to normalize for protein loading.

ADSCs and ADSCKs both maintain AR expression. CAG repeat number in cells at passage 7 did not change in controls (22, 23, 24) and in one SBMA patient (44), increased by one repeat in another patient (45), whereas it increased by 5 repeats in the third patient (51). In RT-qPCR, performed using primers amplifying human AR (Figure 3A) we found that both cell types contained considerable amounts of AR mRNA. However, ADSCKs expressing mutant ARpolyQ were characterized by lower AR mRNA levels when compared to ADSCs expressing wtAR. The reduction of AR expression was similar in all different ADSCKs clones from SBMA patients. This might be due to a negative pressure generated by toxicity of mutant ARpolyQ [28] on patient-derived cells, resulting in clonal selection of cells expressing lower AR levels, thus less sensitive to its toxicity, as also suggested by the decreased ADSCKs growth/survival (Figure 1). WB analysis, performed with anti-AR antibody (Figure 3B), showed that both wtAR and ARpolyQ proteins were correctly translated, being detectable at molecular weights (M.W.) expected for them. As expected, the band of the mutant ARpolyQ had an apparent higher M.W. (slower gel mobility migration) than that of wtAR, because of the presence of the elongated polyQ tract which slightly increases its total mass [3]. In line with our previous observations [28], [29], [30], testosterone stabilized both wt and SBMA AR, thus increasing their intracellular levels, but no relevant amounts of high M.W. SDS-resistant species were observed in the stacking gel. Thus, the ADSC protein quality control system correctly handles potentially misfolded ARpolyQ species induced by testosterone. Even if WB is a qualitative analysis, the levels of mutant ARpolyQ appeared lower than that of wtAR (as seen with quantitative RT-qPCR analysis).

### Biochemical behaviour of wtAR and SBMA ARpolyQ in ADSCs and ADSCKs

**Figure 4:**
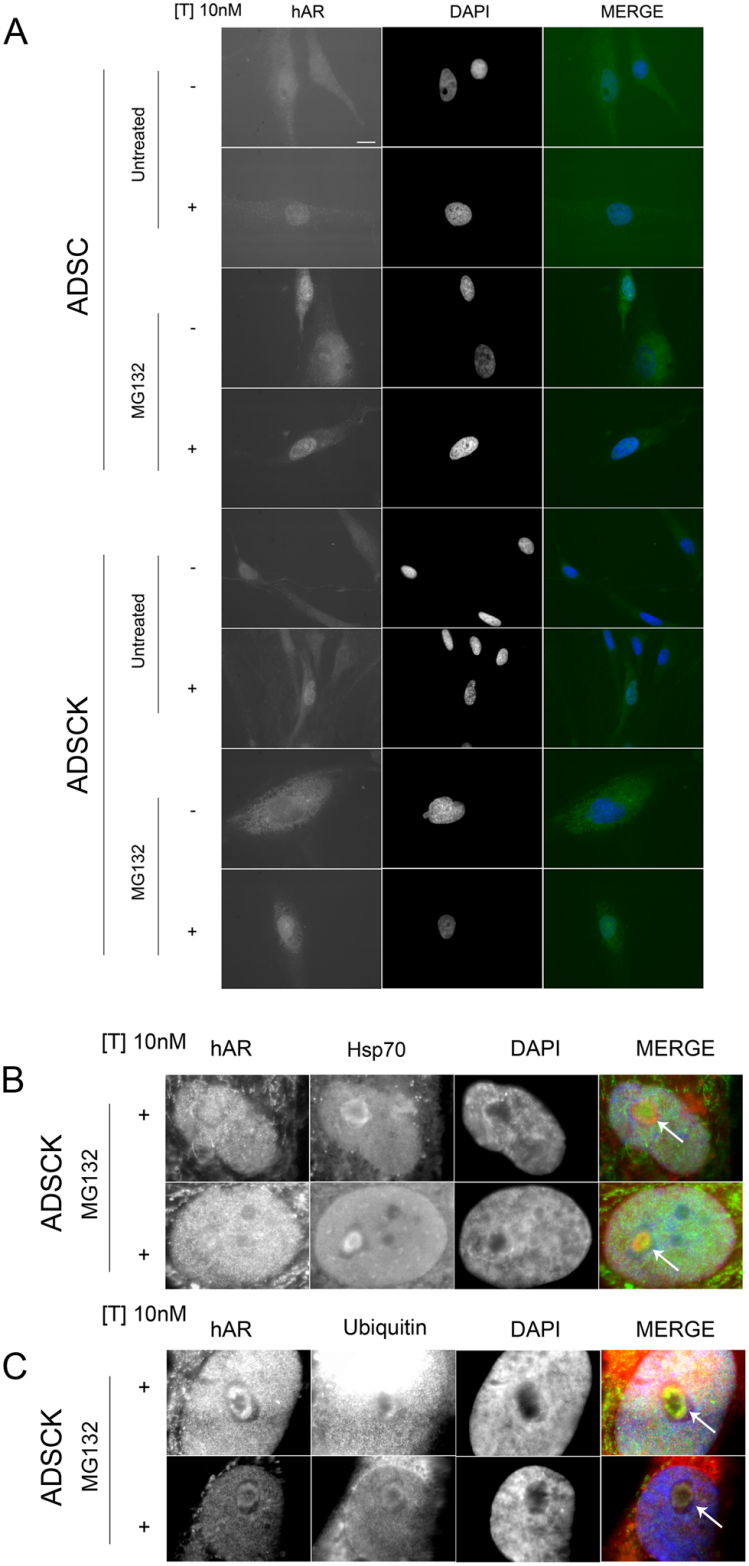
Biochemical behaviour of AR in ADSCs and ADSCKs. (A) High resolution fluorescence microscopy analysis (63X magnification) performed on ADSCs and ADSCKs in absence (–T) or in presence (+T) of 10 nM of testosterone for 48 hours in basal condition or after treatment with 10 µM of MG132 for 24 hours. Nuclei were stained with DAPI. Scale bar 10 µm. (B) High resolution fluorescence microscopy analysis (63X magnification) performed on ADSCKs in the presence (+T) of 10 nM of testosterone for 48 hours after treatment with 10 µM of MG132 for 24 hours. Fluorescence microscopy localization of AR (green) and Hsp70 (red). Nuclei were stained with DAPI. (C) High resolution fluorescence microscopy analysis (63X magnification) performed on ADSCKs in presence (+T) of 10 nM of testosterone for 48 hours after treatment with 10 µM of MG132 for 24 hours. Fluorescence microscopy localization of AR (green) and ubiquitin (red). Nuclei were stained with DAPI. Panel B and C represent only the nuclear area of the cells where the aggregates were observed. The arrows indicate the ARpolyQ inclusions co-stained with Hsp70 and ubiquitin, respectively.

To evaluate whether testosterone normally induces wtAR or ARpolyQ translocation into the nuclei of ADSCs, we performed immunofluorescence analysis using an anti-AR antibody. Figure 4A shows that without testosterone both ARs were cytoplasmic; testosterone treatment (10 nM) for 48 hours induced complete wtAR translocation to the nucleus, while ARpolyQ did not completely translocate to the nucleus, as a small amount remained cytoplasmic. When we evaluated the effect of proteasome blockage on AR clearance, we found that treatment with proteasome inhibitor MG132 induced an overall increase of both wtAR and ARpolyQ levels in cytoplasm and nucleus. Interestingly, we noted that in ADSCKs the co-treatment with testosterone and MG132 induced formation of ARpolyQ nuclear inclusions in a few cells (approximately 5% of total analysed cells); the autophagy inhibitor 3-MA increased ARpolyQ levels, but did not induce intranuclear inclusions (not shown). Thus, the proteasome appears to be one of the preferential pathways to clear the misfolded fraction of ARpolyQ, capable to aggregate, present in ADSCKs. Therefore, we analysed whether MG132-induced ARpolyQ nuclear inclusions directly resulted from inhibition of degradative systems, by analysing possible sequestration of two proteins essential for proteasomal degradation. We found that testosterone- and MG132-induced nuclear inclusions sequestered the chaperone HSP70 (responsible for recognition of misfolded protein species to be degraded by ubiquitin-proteasome pathway) (Figure 4B) and were also positive for ubiquitin (Figure 4C). This suggests that some misfolded species of ARpolyQ present in ADSCKs require a functional proteasome for their clearance, even if an indirect effect can also be postulated. In fact, several stressor stimuli resulting from proteasome blockage (which limits the clearance also of other ubiquitinated proteins) are added to a system, which is possibly almost overwhelmed by the presence of mutant ARpolyQ.

Collectively, these data demonstrate that ADSCKs cells are valuable tool to model pathogenic events taking place in cells of SBMA patients.

## Discussion

In this study we explored the use of adipose-derived stem cells isolated from periumbilical fat as a new human *in vitro* model to investigate AR behaviour and toxicity in SBMA.

Our results demonstrate that the isolation and expansion of ADSCs from healthy volunteers and from SBMA patients is feasible, and the different cell populations obtained from three controls and three SBMA patients were comparable morphologically. However, the growth capacity of ADSCKs was significantly lower than ADSCs, evidenced by a reduced survival rate of ADSCKs as compared to normal ADSCs. This apparently contrasts a recent study by Huang and colleagues showing that if AR functions are suppressed, murine ADSCs have higher growth rate [31]. In fact, they found that AR plays a suppressive role in self-renewal of the bone marrow MSCs isolated from AR knock-out mice (ARKO), by inactivating Akt and Erk signalling [31]. It is possible that the reduced growth rate and lower AR expression of mutant cells is due to selective pressure caused by polyQ toxicity, although this idea remains to be explored. In fact, both cell types express high amounts of AR mRNA and protein, but at different levels, and AR expression is significantly lower in ADSCKs than in ADSCs, in agreement with other studies performed by comparing wt and SBMA cell models [28], [32], [33].

An immunophenotypic marker profile in ADSCs and ADSCKs confirmed their mesenchymal identity as required by guidelines [34] and was maintained by ADSCs in culture at passage 3 and 8. Despite this, ADSCs showed only a mild positivity for CD34, and this might depend on the tissue collection procedure, degree of bleeding, vascular isolation technique or chopping [35]. Conversely, in ADSCKs we observed expression of HLA-DR. In MSC from bone marrow, this marker is induced by interferon-gamma treatment [36], thus an abnormal inflammatory response associated with mutant ARpolyQ could induce HLA-DR expression in ADSCKs, as ADSCs usually do not express this marker.

It is unclear whether the toxic effect of ARpolyQ is also responsible for the different potentiality of ADSCs and ADSCKs, since ADSCs differentiated into all the three cell lineages possibly deriving from MSC (adipocytes, osteocytes and chondrocytes), while ADSCKs only generated adipocytes. We believe that this differentiation inability might depend on mutant ARpolyQ, since ARKO mice have a significant decrease in bone mass, and bone marrow MSCs derived from these mice have a reduced expression of osteogenic markers during the lineage differentiation without affecting the MSC identity [37].

ADSCKs may be used to study pathophysiology of SBMA. In fact, both wt and mutated AR maintained the expected response to their ligand, i.e., when exposed to testosterone AR translocated to the nucleus. Moreover, under particular conditions we could reproduce the formation of inclusions, a specific (though possibly protective) cellular reaction in SBMA, as well as in other misfolded protein diseases. Indeed, we observed that when proteasome function is pharmacologically blocked, a fraction of ARpolyQ (normally cleared via this pathway) accumulates into ubiquitinated HSP70-positive nuclear inclusions, similar to those usually present in spinal cord motoneurons of SBMA patients. This confirms that proteasome may be responsible for ARpolyQ clearance, and demonstrates that our novel cellular model recapitulates a typical feature present in the tissue of affected SBMA individuals. However, it is also possible that our stressor stimuli linked to proteasome blockage, which also impairs the clearance of other ubiquitinated proteins, when added to a system which is possibly almost overwhelmed by the presence of mutant ARpolyQ, may limit the clearance of all the misfolded species including ARpolyQ.

Interestingly, SBMA patient-derived iPSCs have been recently used to study some molecular alteration occurring as a consequence of ARpolyQ expression. The great advantage of these SBMA iPSCs is to be of human origin, and with a great potential to be differentiated to “bona fide” motoneurons [19]. Using the iPSCs models, Grunseich and coll. showed that testosterone activated ARpolyQ induced an increase of acetylated alpha-tubulin and reduced HDAC6 [19], with consequently a reduction of the perinuclear accumulation of lysosomes.

A limitation of the iPSCs could be that, as far as we know, there are no established procedures to differentiate them into muscle cell types. In our view, this is relevant, since muscle cells have been recently recognized as targets of ARpolyQ toxicity [17], [38], [39]. However, to the best of our knowledge, all attempts to generate muscle cells from iPSCs failed so far. In addition, iPSCs are produced by genetic transformation of fibroblasts, using four oncogenic or differentiating agents that may impact on cell behavior. Thus, other cell models of human origin may be of value to complement the data obtained in iPSCs. Conversely, despite the fact that ADSCKs are difficult to maintain in culture for many passages, there are studies demonstrating the possibility to differentiate ADSC into muscle cell lines [40]–[43]. It must be taken into account also that, compared to iPSCs, ADSCs have the advantages of no retro transduction and manipulation needing, and thus do not express exogenous genes.

In conclusion, ADSCs represent a potential novel model of patient-derived cell populations useful to study the SBMA disease mechanism. Although these cells are not differentiable in motoneurons, at present, ADSCKs express AR and mimic some pathogenic SBMA mechanisms. Moreover, ADSCKs can be easily obtained with minimally invasive approach. Therefore, they have an interesting and still unexplored potential in studying disease mechanisms, and in designing and testing therapeutic approaches in SBMA and other disorders.
